# OA25 The formation of The CTD Service at Sheffield Children’s Hospital

**DOI:** 10.1093/rap/rkag113.025

**Published:** 2026-11-03

**Authors:** Sarah Maltby, Sameena Khalid, Anne-Marie Mcmahon, Lizzie Deugo, Maysoon Elfadil

**Affiliations:** Sheffield Children’s Hospital, Sheffield, United Kingdom; Sheffield Teaching Hospital, Sheffield, United Kingdom; Sheffield Children’s Hospital, Sheffield, United Kingdom; Sheffield Children’s Hospital, Sheffield, United Kingdom; Sheffield Children’s Hospital, Sheffield, United Kingdom

## Abstract

**Introduction:**

The case of a sick lupus patient shaped to the formation of Sheffield Children’s Hospital Connective Tissue Disease (CTD) service. An urgent Multidisciplinary Team (MDT) meeting with adult CTD experts highlighted that we were national outlier, with a lack of a dedicated CTD service. Over the last three years we have developed the CTD service, modelled on the successful, nationally recognised Sheffield Adolescent Service. We currently provide monthly joint MDT clinics with adult colleague, two monthly joint dermatology clinics and monthly MDT meetings. This has enabled timely reviews, safe monitoring and a robust transition process for our most complex patients.

**Case description:**

A twelve year old girl, previously fit and well, presented with rapidly progressing arthritis of multiple joints and pyrexia. Investigations revealed ESR 94, CRP 30, ANA positive, double stranded DNA >379, and she was diagnosed with Juvenile Systemic Lupus Erythematosus (JSLE). She was treated with steroids, Methotrexate and Hydroxychloroquine and a marked clinical improvement was noted initially, with normal inflammatory markers at three months post diagnosis.

She presented Six months later, having been on and off treatment due to upper respiratory tract infections, with lethargy, joint pain, hair loss and a malar rash (double stranded DNA >379). She was treated for lupus flare and pulsed with Intravenous methylprednisolone. Immediately following this she became acutely unwell, with pyrexia and respiratory distress, and rapidly deteriorated, requiring escalation to intensive care and ventilation. Her chest x-ray showed widespread bilateral changes. Further investigations including bronchoalveolar lavage did not reveal an obvious infective cause. She was however treated for infection and inflammation with antibiotics and steroids, and gradually started to recover, and she was finally extubated after seven days and rapid clinical improvement was seen. Following regular clinic review her JSLE treatment was optimised to include mycophenolate mofetil and Rituximab, and she remains stable, approaching transition to the adult services.

**Discussion:**

Infection, inflammation or both is the dilemma that rheumatologists face daily, and requires a careful MDT approach. This is paramount when dealing with complex and rare disease such JSLE. In this case we needed to seek urgent advise from Infectious disease, respiratory and adult rheumatology experts, to ensure a safe collaborative patients care. As infection and inflammation drive each other, treating for both is often required.

This case highlighted the need for timely reviews and a close monitoring system for these complex patients to prevent future deteriorations before they occur. The CTD service in Sheffield has grown from this case with regular MDT clinics to monitor such patients, as well as regular MDT meetings to discuss patients in detail and optimise care. We are also involved in local and national CTD research, to enable evidenced based care to progress our service and knowledge further.

The trauma of being on intensive care and the realisation of the seriousness of her disease, as well as starting adolescence, really impacted this patients physical and mental health. The CTD service has enabled a holistic approach to with psychology, physiotherapy and occupational therapy input, she has returned to school and normality.

The CTD service has provided her with early adolescent rheumatology introduction, with adult rheumatologist regular input. CTD is much more common in adults than children and this model is crucial to deliver the best care to our patients. This input has enabled a robust transition of her care to the adult services and she is feeling positive about her future.

**Key learning points:**

This case highlighted the following key learning points;

1. Complex inflammation can lead to rapid deterioration and needs careful monitoring

2. Inflammation and infection drive each other, in uncertainty, use a collaborative approach and consider treating for both

3. Complex CTD patients require a multidisciplinary approach. Timely reviews and regular monitoring are key

4. Use all members of the MDT to provide holistic patient care

5. Early adolescent input enables robust transition

